# Psychological correlates of COVID-19 pandemic in the Austrian population

**DOI:** 10.1186/s12889-020-09489-5

**Published:** 2020-09-14

**Authors:** Claudia Traunmüller, Rene Stefitz, Kerstin Gaisbachgrabner, Andreas Schwerdtfeger

**Affiliations:** Institute of Psychology, Health Psychology Unit, University of Graz, 8010 Graz, Austria

**Keywords:** COVID-19 pandemic, Stress, Anxiety, Depression, Psychological status

## Abstract

**Background:**

COVID-19 poses the greatest challenge for the entire world since the Second World War. Governments are forced to define strict measures to avoid the spreading of the virus, which may further impose psychological burden for the majority of the population. The aim of this study was to evaluate the psychological distress in Austria during the initial stage of the COVID-19 outbreak.

**Methods:**

From 25 March to 3 April 2020, an anonymous online survey was conducted. Target group included all members of the Austrian population older than 16 years. The survey addressed the following areas (1) and sociodemographic data, (2) physical and mental health; (3) knowledge and concerns about COVID-19; (4) contact with infected people; (5) prevention efforts; (6) need for further information. The Impact of Event Scale-Revised (IES-R) and the Depression, Anxiety and Stress Scale (DASS-21) were used to assess mental health. Analyses were based on 4126 individuals (74% female, age: M = 38.68, SD = 13.36).

**Results:**

43.3% rated the psychological impact as moderate (5.6%) or severe (37.7%). 26.5% reported moderate (13.3%) to severe (13.2%) depression; 20.3% moderate (8.9%) to severe (11.4%) anxiety and 21.2% reported to suffer from moderate (10.5%) or severe stress (10.7%). Being female, higher age, lower levels of education, concern about family members, internet as main source of information, student or pupil status, poor self-rated health, and downplaying the seriousness of the problem were significantly associated with higher psychological burden. Protective factors were the possibility to work in home office, frequent (indirect) contact with family or friends, the availability of virus-specific information, confidence in the diagnosis capability, and physical activity during the crisis.

**Conclusion:**

This study is among the first in Europe on the psychological correlates of the COVID-19 pandemic. 37.7% of the Austrian study population reported a severe psychological impact on the event and 1 in 10 is considered to suffer from severe depression, anxiety or stress. The present findings inform about the identification of protective factors, psychologically vulnerable groups and may guide the development of psychological interventions.

## Background

Severe acute respiratory syndrome coronavirus 2 (SARS-CoV-2) was identified first on January 10 in Wuhan, China. This virus triggers the COVID-19 pandemic, which was classified by the WHO on January 30, 2020, as a health emergency of international scope (https://www.who.int/westernpacific/emergencies/covid-19). In the meantime, the whole world has been affected by this virus with unforeseeable economic, personnel and psychological consequences.

COVID-19 virus is a highly contagious disease. The fundamental challenge of such a pandemic is that not every person shows the same symptoms. It is supposed that people can be infected with this virus without showing any typical symptoms, making a reliable estimate about its prevalence difficult. The finding that the reproductive number of the COVID-19 virus has been estimated at 4.08 – meaning that on average, every case of COVID-19 will create up to 4 new cases – stresses the need for the implementation of strict policies [1]. Similar to other virus epidemics like, for example, SARS, people in the surroundings can be infected through sneezing and coughing or even merely through speaking [2]. According to the European Union (https://www.ecdc.europa.eu/en/geographical-distribution-2019-ncov-cases), there were 786,459 confirmed COVID-19 cases worldwide, including 9634 in Austria, and 37,831 deaths worldwide, and 108 deaths in Austria by March 30, 2020. At the same stage of development of the outbreak China reported 81,518 cases and 3305 deaths (https://www.worldometers.info/coronavirus/country/china/).

**Fig. 1:**
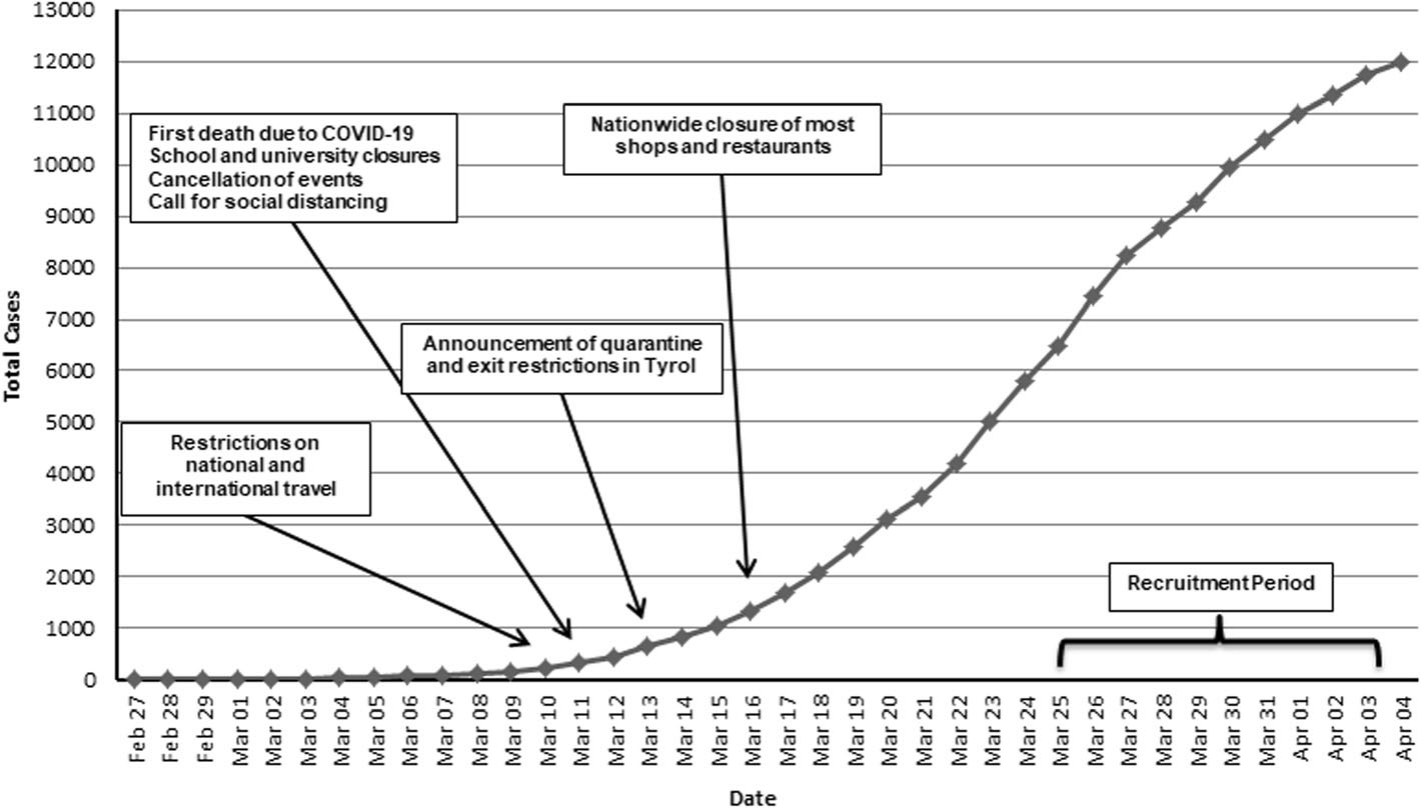
National epidemic trend of COVID-19 outbreak in Austria from 27 February to 4 April 2020

Based on these data, many governments worldwide defined strict restrictions to reduce the risk of new infections within the population and to protect the health care system from excessive demands. Experiences from previous virus epidemics showed that the uncontrolled spread of a virus can only be prevented by instituting widespread and strict quarantine policies with large personal restrictions [3]. On February 25, Austrian authorities reported the first COVID-19 in Austria and on March 11 the first death due to this virus infection. Since that time, numbers of infections and deaths have increased exponentially in Austria and all over the world. Northern Italy, in particular, was severely affected by the COVID-19 pandemic and reported hundreds of deaths a day. Due to its proximity to Italy, some Austrian provinces of Tyrolia has been declared a quarantine zone on March 13. Three days after that, the Austrian authorities imposed measures with strict personal recommendations against the spreading of the COVID-19 virus, which included a complete shutdown of the industry (except industries necessary for the immediate supply of the population, like pharmacies, hospitals and groceries) and strict personnel restrictions. Apart from preventive hygiene measures like washing the hands or sneezing in the elbow, the Austrian government imposed a complete curfew with some exceptions: (a) shopping for food or medication, (b) helping other people, (c) go to work or (d) to take a walk or do sports. Individuals are encouraged to keep a distance of 2 m to other people. It was generally forbidden to meet other people who do not live in the same household. The whole country of Austria has been in a historical state of emergency. The Austrian government and politicians speak of circumstances that have not existed since the Second World War. Figure 1 shows the development of the COVID-19 outbreak, the measures taken to control the spread of the virus and the recruitment period of the participants.

Although these measures were successful in slowing down the outbreak in all areas of the country, there is evidence that quarantine policies create psychological, emotional, and financial problems for the majority of the population [3]. A recent review of Brooks et al. (2020) pointed out the negative psychological effects of quarantine. It can be assumed, that such measures pose a burden on individuals, thus challenging mental health and resilience [4].

Based on the study of Wang et al. (2020), the present study is the first nationwide large-scale survey of the psychological correlates and mental health in Austria during the initial state of COVID-19 pandemic. Results may inform about the psychological needs of the population and could provide a basis for tailored mental health interventions in order to support the population in this exceptional situation. Therefore, the main focus of the study was on the evaluation of the extent of psychological distress of the Austrian population and to contribute to international comparisons regarding the mental health correlates of the COVID-19 pandemic.

## Methods

### Participants

A cross-sectional study design was implemented to assess the potential psychological distress during the COVID-19 pandemic in the Austrian population within the first 2 weeks after implementation of strict personnel restrictions, by using an anonymous online survey (LimeSurvey GmbH, Hamburg, Deutschland). Target group included all members of the public exceeding the age of 16 years. The online survey was distributed in social and print media with a request to pass it on to others. A total of 4618 people answered the questionnaire. Four hundred ninety two individuals had to be excluded due to incorrect completion (e.g., not fully completed). Analysis was based on 4126 individuals (women: *N* = 3042, men: *N* = 1073; age: *M* = 38.68, *SD* = 13.36, Range: 16 years – 82 years).

### Procedure

Data collection took place over 10 days (25 March – 3 April 2020), after the Austrian government imposed measures with strict personal recommendations against the spreading of the COVID-19 virus. These measures were implemented 14 days after the first COVID-19 infection in Austria were detected (see Fig. 1).

### Survey development

Based on the methodological approach of Wang et al. (2020), several instruments were used to address the following areas, in addition to sociodemographic data: (1) physical health status (currently, and over the past 14 days); (2) basic knowledge and concerns about COVID-19; (3) contact with COVID-19 infected people in the past 14 days; (4) prevention measures against COVID-19 in the past 14 days; (5) additional information required with respect to COVID-19; and (6) the psychological correlates of the COVID-19 outbreak.

A number of sociodemographic data were gathered (age, sex, relationship status, educational level, current employment status). Furthermore, individuals were asked about their current work situation, for example, if they lost their job because of the COVID-19 pandemic or how they organized their everyday work at home, how many hours they spent at home on average and how often they had contact with people outside the household.

Specific questions concerning health status were also evaluated and were always related to the past 14 days. Physical symptoms including fever, headache and muscle pain, coughing shortness of breath, dizziness and sore throat were assessed. Individuals were asked whether they received medical treatment, were hospitalized or had been tested for the COVID-19 virus in the last 14 days. Furthermore, individuals were asked to rate their current physical health status and give additional information about existing chronic illnesses. Self-rated physical health status was measured on a 5-point Likert scale (1 = very poor to 5 = very good). Contact history variables included direct or indirect contact with confirmed COVID-19 individuals as well as contact with materials or individuals with suspected COVID-19.

Furthermore, knowledge about COVID-19 was assessed. The following variables were used: knowledge about transmission pathways (queried via a knowledge test), level of confidence in the current diagnostic options, satisfaction with health information about COVID-19 and information about the development of new infections and deaths. Individuals were asked about their sources from which they obtained the information. Respondents were also asked to what extend they were afraid that they themselves or a family member could become infected with COVID-19. Finally, they were asked about their use of precautionary measures (washing hands, wearing masks, etc.) against COVID-19 infection and how high they rated the chances of surviving a COVID-19 infection. 5-point Likert scales were used to measure participant’s attitudes, opinions and perceptions (e.g., 1 = very unlikely to 5 = very likely). Participants were also requested to indicate whether they were in need of further information regarding treatment, distribution opportunities, local outbreaks, etc.

The Impact of Event Scale-Revised (IES-R; Maercker & Schützwohl, 1998) was used to evaluate the psychological correlates of COVID-19. It is free to use and aims to measure the subjective response to a specific traumatic event [5]. The IES-R is a short and easy self-administered questionnaire with 22 items and three subscales. The main focus of this questionnaire is to measure the mean avoidance, intrusion, and hyperarousal [6]. Furthermore, a total subjective stress IES-R score can be calculated. Referred to Creamer et al. (2003), the sum score can be divided into 0–23 (normal), 24–32(mild psychological impact), 33–36 (moderate psychological impact), and > 37 (severe psychological impact). Originally, this test has been developed for American students, but it has also been used across diverse populations (including different national groups), whereby the factor structure has remained robust [7]. In the present study, reliability for this scale (Cronbach’s alpha = .93) was very good. For analysis the sum score was calculated.

In order to examine the mental health status, the short form of the depression, anxiety and stress-scale (DASS-21, Nilges & Essau, 2015) was used. This inventory is free to use and has been applied in research related to virus-epidemics [8]. DASS-21 is a well-established instrument to measure the core symptoms of depression, anxiety and stress [9]. All subscales consisted of 7 items and the scores of the subscales (depression, anxiety, and stress) were divided into categories from normal to extremely severe. In more detail, for depression: mild depression [7, 8, 10], moderate depression [9, 11–17], severe depression [18–24], and extremely severe depression (28–42); for anxiety: normal (0–6), mild anxiety [5, 6, 25], moderate anxiety [7–11], severe anxiety [12–16], and extremely severe anxiety (20–42), and for stress: normal (0–10), mild stress [8–15], moderate stress [16–23], severe stress [24, 26–32], and extremely severe stress (35–42). In the present study, reliabilities for the subscales depression (Cronbach’s alpha = .91), stress (Cronbach’s alpha = .88) and anxiety (Cronbach’s alpha = .91) were very good. For analysis, the sum scores of the three subscales stress, anxiety and depression were calculated.

### Statistical analysis

Statistical analyses were conducted using the software IBM SPSS statistics version 24 (IBM Corp., NY, USA). The general level of significance was fixed at *p* < .05 (two tailed). Descriptive statistics were calculated for all variables and presented as mean (*M*) and standard deviation (*SD*) for continuous variables, median and percentages or response for categorical and nominal variables. Linear regression was used to analyze univariate association between predictor variables and the sum scores of the DASS-21 subscales and the IES-R. In order to ensure comparability with the results of Wang et al. (2020), reference categories for the linear regression analyses were based on Wang’s methodology. Ordinal data from Likert scales were treated as continuous variables (e.g., frequency of precautionary measures). Chi-Square tests of independence were used to determine if there were significant differences between nominal variables. Bonferroni-Holm procedure was utilized to adjust for multiple comparisons.

## Results

### Survey respondents

Psychological symptoms during of COVID-19 were measured using the DASS-21 [10] and the IES-R [33]. The sample mean score for the DASS-21 scale was *M* = 24.87 (*SD* = 26.97). Sample mean scores for the DASS-21 subscales were 8.88 (*SD* = 10.26) for depression, 5.42 (*SD* = 8.38) for anxiety and 10.58 (*SD* = 10.85) for stress.

According to the cut-off scores proposed by Wang et al. (2020), 2652 (64.3%) participants showed a low score on the depression subscale (score: 0–9), 383 (9.3%) were considered to suffer from mild depression (score: 10–12), 547 (13.3%) from moderate depression (score: 13–20), and 544 (13.2%) from severe or extremely severe depression (score: 21–42). *N* = 3069 (74.4%) participants showed a low score on the anxiety subscale (score: 0–6), 216 (5.2%) were considered to suffer from mild anxiety (score: 7–9), 369 (8.9%) from moderate anxiety (score: 10–14), and 472 (11.4%) from severe or extremely severe anxiety (score: 15–42). *N* = 2546 (61.7%) participants showed a low score on the stress subscale (score: 0–10), 703 (17%) were considered to suffer from mild stress (score: 11–18), 435 (10.5%) from moderate stress (score: 19–26), and 442 (10.7%) from severe or extremely severe stress (score: 27–42).

Cut-off scores from the German version of the DASS-21 [10] revealed 544 (13.2%) clinically relevant cases of depression, 574 (13.9%) clinically relevant cases of anxiety and 769 (18.6%) clinically relevant cases of stress.

The sample mean score of the IES-R was 32.36 (*SD* = 24.02). According to the cut-of scores proposed by Creamer et al. (2003), 1771 (42.9%) participants rated the psychological impact as minimal (score < 23), 570 (13.8%) as mild (score: 24–32), 231 (5.6%) as moderate, and 1554 (37.7%) as severe (score: > 33).

In direct comparison with the results of Wang et al. (2020), significantly less Austrian individuals rated the psychological impact of the COIVD-19 outbreak as moderate or severe (Wang et al., 2020: 53.8% vs 43.3%; *X*^*2*^ (2, *N* = 5336) = 142.67, *p < .*001. In contrast, significantly more participants of the Austrian sample reported severe depression (Wang et al., 2020: 4.3% vs 13.2%; *X*^*2*^ (3, *N* = 5336) = 89.61, *p < .*001), severe anxiety (Wang et al., 2020: 8.4% vs 11.4%; *X*^*2*^ (3, *N* = 5336) = 138.02, *p < .*001) and severe stress (Wang et al., 2020: 2.6% vs 10.7%; *X*^*2*^ (3, *N* = 5336) = 126.79, *p < .*001).

### Associations with sociodemographic variables

Men reported significantly lower stress (*B* = − 4.68, 95% *CI* = − 5.42, − 3.94, *p < .*001), anxiety (*B* = − 2.59, 95% *CI* = − 3.17, − 2.01, *p < .*001), depression (*B* = − 2.74, 95% *CI* = − 3.45, − 2.03, *p < .*001), and less psychological impact of the current event (*B* = − 11.41, 95% *CI* = − 13.04, − 9.78, *p < .*001) than women. Higher age was significantly associated with less stress (*B* = − 0.11, 95% *CI* = − 0.13, − 0.09, *p < .*001), anxiety (*B* = − 0.05, 95% *CI* = − 0.08, − 0.03, *p < .*001), and depression (*B* = − 0.11, 95% *CI* = − 0.13, − 0.09, *p < .*001). Participants living together with children showed significantly higher stress (*B* = 0.82, 95% *CI* = − 1.83, − 0.51, *p < .*001), but lower scores in depression (*B* = − 1.17, 95% *CI* = − 1.83, − 0.51, *p < .*001). Lower levels of education were significantly associated with higher scores of stress, anxiety, depression and psychological impact of COVID-19. For example, lower secondary education was significantly associated with more anxiety (*B* = 2.20, 95% *CI* = 0.63, 3.77, *p = .*006), depression (*B* = 4.33, 95% *CI* = 2.41, 6.25, *p < .*001) and more psychological impact (*B* = 7.59, 95% *CI* = 3.08, 12.10, *p < .*001) compared to doctorate/PhD (for further details, see Table 1). Unemployed status was significantly associated with higher stress (*B* = 3.19, 95% *CI* = 1.61, 4.71, *p < .*001), anxiety (*B* = 2.73, 95% *CI* = 1.55, 3.91, *p < .*001), depression (*B* = 4.60, 95% *CI* = 3.17, 6.03, *p < .*001) and greater impact of the event (*B* = 4.71, 95% *CI* = 1.32, 8.1, *p = .*006) as compared to respondents who were employed. Student, respectively pupil status was significantly associated with higher stress (Student: *B* = 1.83, 95% *CI* = 0.86, 2.80, *p < .*001) and depression (Student: *B* = 3.52, 95% *CI* = 2.61, *p < .*001, 4.43; Pupil: *B* = 5.17, 95% *CI* = 3.26, 7.08, *p < .*001) as compared to employed participants. Individuals working from home showed significantly lower anxiety (*B* = − 1.31, 95% *CI* = − 1.88, − 0.74, *p < .*001), depression (*B* = − 2.28, 95% *CI* = − 2.98, − 1.58, *p < .*001), and IES-R scores (*B* = − 2.34, 95% *CI* = − 3.99, − 0.69, *p = .*005) as compared to those working under normal conditions. Participants on sick leave scored significantly higher in stress (*B* = 3.96, 95% *CI* = 1.51, 6.41, *p = .*002), anxiety (*B* = 4.80, 95% *CI* = 2.91, 6.69, *p < .*001), depression (*B* = 4.17, 95% *CI* = 1.86, 6.48, *p < .*001), and IES-R (*B* = 8.93, 95% *CI* = 3.5, 14.36, *p = .*001) as compared to those working under normal conditions. Further analyses revealed that an increase of hours per day spent at home was significantly associated with higher stress (*B* = 0.19, 95% *CI* = 0.11, 0.27, *p < .*001), anxiety (*B* = 0.10, 95% *CI* = 0.04, 0.16, *p = .*002) and depression (*B* = 0.18, 95% *CI* = 0.11, 0.25, *p < .*001), while a higher number of social contacts with friends and/or family members was significantly associated with lower scores in anxiety and depression. For example, contacting family or friends every few days was significantly associated with lower anxiety (*B* = − 3.51, 95% *CI* = − 5.66, − 1.36, *p = .*001) and depression (*B* = − 3.69, 95% *CI* = − .33, − 1.05, *p = .*006) as compared to no contact. For further details, see Table 1.

**Table 1:** Linear regression results of demographic variables associated with the psychological impact of the 2020 COVID-19 outbreak measured with DASS-Subscales and the IES-R

***Variables***	***N (***%***)*** ***M (SD)***	Stress			Anxiety			Depression			Impact of Event		
		*R*^*2*^	*B (*95% *CI)*	*p*	*R*^*2*^	*B (*95% *CI)*	*p*	*R*^*2*^	*B (9*5% *CI)*	*p*	*R*^*2*^	*B (*95% *CI)*	*p*
**Gender**													
Male	1073 (26)	0.036	4.68 ± 0.74	*<.*001	0.018	−2.59 ± 0.58	*<.*001	0.014	−2.74 ± 0.71	*<.*001	0.043	−11.41 ± 1.63	*<.*001
Female (Ref.)	3042 (74)		–	–		–	–		–	–		–	–
**Age** (Continuous)	38.68 (13.36)	0.018	0.11 ± 0.02	*<.*001	0.006	−0.05 ± 0.02	*<.*001	0.022	− 0.11 ± 0.02	*<.*001	0.000	−0.02 ± 0.05	.48
**Marital status**													
Single	1057 (25.6)		3.37 ± 3.19	*.*039		−0.71 ± 2.47	.58		3.19 ± 3.01	*.*038		4.61 ± 7.09	.20
Married/Partnership	2838 (68.8)	0.002	2.92 ± 3.15	.07	0.001	−1.29 ± 2.44	.30	0.014	0.37 ± 2.97	.80	0.000	4.67 ± 7.00	.19
Divorced	185 (4.5)		1.23 ± 3.49	.49		−1.83 ± 2.71	.19		0.38 ± 3.29	.82		5.54 ± 7.76	.16
Widowed (Ref.)	46 (1.1)		–	–		–	–		–	–		–	–
**Household size** (Continuous)	2.39 (2.21)	0.000	0.03 ± 0.15	.67	0.000	−0.01 ± 0.12	.86	0.000	−0.10 ± 0.14	.16	0.000	0.04 ± 0.33	.80
**Children in household**													
Yes	1448 (35.1)	0.001	0.82 ± 0.69	*.*020	0.000	0.09 ± 0.54	.75	0.003	−1.17 ± 0.66	*<.*001	0.000	1.25 ± 1.54	.11
No (Ref.)	2678 (64.9)		–	–		–	–		–	–		–	–
**Education Level**													
No formal education	31 (0.8)		2.40 ± 4.07	.25	0.007	2.07 ± 3.14	.20	0.013	4.19 ± 3.84	*.*033	0.003	4.31 ± 9.02	.35
Lower secondary education	216 (5.2)		2.60 ± 2.03	*.*012		2.20 ± 1.57	*.*006^a^		4.33 ± 1.92	*<.*001^a^		7.59 ± 4.51	*<.*001^a^
Vocational education	694 (16.8)		0.79 ± 1.64	.35		1.83 ± 1.27	*.*005^a^		1.71 ± 1.55	*.*031		5.21 ± 3.64	*.*005^a^
High School Certificate	1286 (31.2)	0.003	1.66 ± 1.55	*.*036		1.43 ± 1.20	*.*002		2.53 ± 1.46	*<.*001^a^		3.58 ± 3.44	*.*041
Bachelor Degree	584 (14.2)		1.33 ± 1.68	.12		0.87 ± 1.30	.19		1.70 ± 1.58	*.*036		2.87 ± 3.73	.13
Master Degree	1096 (26.6)		0.91 ± 1.57	.26		0.11 ± 1.21	.86		0.25 ± 1.48	.74		2.20 ± 3.48	.22
Doctorate/PhD (Ref.)	219 (5.3)		–	–		–	–		–	–		–	–
**Employment status**		0.010			0.007			0.027			0.003		
Unemployed	208 (5)		3.19 ± 1.52	*<.*001^a^		2.73 ± 1.18	*<.*001^a^		4.60 ± 1.43	*<.*001^a^		4.71 ± 3.39	*.*006^a^
Retired	343 (8.3)		−1.40 ± 1.21	*<.*05		0.46 ± 0.94	.34		0.14 ± 1.14	.81		2.62 ± 2.70	.06
Student	586 (14.2)		1.83 ± 0.97	*<.*001^a^		0.91 ± 0.75	*.*018		3.52 ± 0.91	*<.*001^a^		−0.04 ± 2.15	.97
Pupil	113 (2.7)		1.98 ± 2.03	.06		1.49 ± 1.58	.06		5.17 ± 1.91	*<.*001^a^		1.39 ± 4.52	.55
Self-Employed	287 (7)		−0.74 ± 1.31	.27		−0.51 ± 1.02	.33		−0.63 ± 1.23	.32		−1.86 ± 2.93	.21
Employed (Ref.)	2589 (62.7)		–	–		–	–		–	–		–	–
**Daily working routine only (Self-)Employed (*N*** **= 2876)**													
Home Office	1438 (50)		−0.71 ± 0.74	.06		−1.31 ± 0.57	*<.*001^a^		−2.28 ± 0.70	*<.*001^a^		−2.34 ± 1.65	*.*005^a^
Holiday	153 (5.3)		0.12 ± 1.78	.89		−0.32 ± 0.77	.63		−1.09 ± 1.68	.20		−2.03 ± 3.95	.31
Sick Leave	78 (2.7)	0.004	3.96 ± 2.45	*.*002^a^	0.012	4.80 ± 1.89	*<.*001^a^	0.015	4.17 ± 2.31	*<.*001^a^	0.005	8.93 ± 5.43	*.*001^a^
Others	588 (20.4)		−0.08 ± 1.00	.87		−0.32 ± 0.77	.41		−1.41 ± 0.94	*.*003^a^		0.10 ± 2.22	.93
Normal (Ref.)	619 (21.5)		–	–		–	–		–	–		–	–
**Hours per day spent at home (Continuous)**	21.22 (4.17)	0.005	0.19 ± 0.08	*<.*001	0.002	0.10 ± 0.06	*.*002	0.005	0.18 ± 0.07	*<.*001	0.001	0.13 ± 0.18	.14
**Contact to family/friends outside**													
Several times a day	2270 (55)		−1.21 ± 2.71	.38		−2.52 ± 2.10	*.*018*		−3.23 ± 2.57	*.*014^a^		−1.30 ± 6.01	.67
Daily	888 (21.5)		−1.47 ± 2.77	.30		−2.87 ± 2.14	*.*009^a^		−3.23 ± 2.62	*.*016^a^		−2.50 ± 6.13	.42
Every few days	762 (18.5)	0.002	−2.29 ± 2.78	.11	0.004	−3.51 ± 2.15	*.*001^a^	0.003	−3.69 ± 2.64	*.*006^a^	0.003	−4.58 ± 6.17	.15
Once in a Week	143 (3.5)		−1.54 ± 3.21	.35		−1.64 ± 2.48	.20		−2.87 ± 3.04	.06		−1.87 ± 7.11	.61
Never (Ref.)	63 (1.5)		–	–		–	–		–	–		–	–

*Notes.* Descriptive statistics are presented as mean and standard deviations in parentheses for continuous predictor variables or n (%) for categorical predictor variables

^a^ = significant after Bonferroni Holm adjustment for multiple comparisons

### Associations with health status

Higher scores in self-rated health were significantly associated with lower scores in stress (*B* = − 4.83, 95% *CI* = − 4.39, − 5.27, *p < .*001), anxiety (*B* = − 3.76, 95% *CI* = − 3.42, − 4.10, *p < .*001), depression (*B* = − 4.83, 95% *CI* = − 4.39, − 5.27, *p < .*001), and IES-R (*B* = − 7.68, 95% *CI* = − 6.67, − 8.69, *p < .*001). Several participants reported a range of physical symptoms, most frequently headache (46.7%), coryza (31.7%), sore throat (23.5%), myalgia (22.7%), cough (21.5%), dizziness (15.3%), respiratory problems (5.4%), chills (3.6%), and fever (1.8%). Linear regression analyses showed that physical health concerns were significantly associated with higher scores in stress, anxiety, depression and the IES-R scores. For example, having a pre-existing health condition was significantly associated with more stress (*B* = 1.83, 95% *CI* = 0.99, 2.67, *p < .*001), anxiety (*B* = 2.10, 95% *CI* = 1.45, 2.75, *p < .*001), depression (*B* = 1.67, 95% *CI* = 0.88, 2.46, *p < .*001) and higher scores in the IES-R (*B* = 4.26, 95% *CI* = 2.40, 6.12, *p < .*001). For further details, see Table 2.

**Table 2:** Linear regression results of physical health variables associated with the psychological impact of the 2020 COVID-19 outbreak measured with DASS-Subscales and the IES-R

***Variables***	***N (%)*** ***M (SD)***	Stress			Anxiety			Depression			Impact of Event		
		*R*^*2*^	*B (*95% *CI)*	*p*	*R*^*2*^	*B (*95% *CI)*	*p*	*R*^*2*^	*B (*95% *CI)*	*p*	*R*^*2*^	*B (*95% *CI)*	*p*
**Subjective health status**													
**5-point Likert scale**	4.24 (0.71)	0.100	−4.83 ± 0.44	*<.*001	0.100	−3.76 ± 0.34	*<.*001	0.095	−4.46 ± 0.42	*<.*001	0.051	−7.68 ± 1.01	*<.*001
(Continuous)			–	–		–	–		–	–		–	–
**Pre-existing condition**													
Yes	794 (19.2)	0.004	1.83 ± 0.84	*<.*001	0.020	2.10 ± 0.65	*<.*001	0.004	1.67 ± 0.79	*<.*001	0.005	4.26 ± 1.86	*<.*001
No (Ref.)	3332 (80.8)		–	–		–	–		–	–		–	–
**Fever**													
Yes	75 (1.8)	0.001	2.70 ± 2.47	*.*032	0.003	3.17 ± 1.91	*.*001	0.001	2.40 ± 2.34	*.*045	0.002	7.24 ± 5.48	*.*010
No (Ref.)	4051 (80.8)		–	–		–	–		–	–		–	–
**Chills**													
Yes	149 (3.6)	0.018	7.77 ± 1.76	*<.*001	0.026	7.30 ± 1.35	*<.*001	0.014	6.40 ± 1.67	*<.*001	0.011	13.36 ± 3.91	*<.*001
No (Ref.)	3977 (96.4)		–	–		–	–		–	–		–	–
**Headache**													
Yes	1925 (46.7)	0.056	5.15 ± 0.64	*<.*001	0.034	3.09 ± 0.50	*<.*001	0.042	4.24 ± 0.61	*<.*001	0.017	6.27 ± 1.46	*<.*001
No (Ref.)	2201 (53.3)		–	–		–	–		–	–		–	–
**Myalgia**													
Yes	936 (22.7)	0.020	3.62 ± 0.78	*<.*001	0.022	2.97 ± 0.60	*<.*001	0.018	3.27 ± 0.74	*<.*001	0.006	4.42 ± 1.75	*<.*001
No (Ref.)	3190 (77.3)		–	–		–	–		–	–		–	–
**Cough**													
Yes	886 (21.5)	0.006	1.97 ± 0.80	*<.*001	0.008	1.87 ± 0.62	*<.*001	0.002	1.23 ± 0.76	*.*002	0.003	3.19 ± 1.78	*<.*001
No (Ref.)	3240 (78.5)		–	–		–	–		–	–			–
**Respiratory problems**													
Yes	222 (5.4) 3904 (94.6) (5.4)	0.016	1.97 ± 0.80	*<.*001	0.050	8.32 ± 1.11	*<.*001	0.013	5.22 ± 1.38	*<.*001	0.008	9.64 ± 3.24	*<.*001
No (Ref.)	3940 (94.6)		–	–		–	–		–	–		–	–
**Dizziness**													
Yes	631 (15.3)	0.053	6.90 ± 0.89	*<.*001	0.070	6.14 ± 0.69	*<.*001	0.047	6.19 ± 0.85	*<.*001	0.025	10.54 ± 2.01	*<.*001
No (Ref.)	3495 (84.7)		–	–		–	–		–	–		–	–
**Coryza**													
Yes	1307 (31.7)	0.007	1.89 ± 0.71	*<.*001	0.006	1.41 ± 0.55	*<.*001	0.002	1.05 ± 0.67	*.*002	0.000	1.15 ± 1.58	.15
No (Ref.)	2819 (68.3)		–	–		–	–		–	–		–	–
**Sore throat**													
Yes	968 (23.5)	0.019	3.53 ± 0.77	*<.*001	0.021	2.85 ± 0.60	*<.*001	0.008	2.11 ± 0.74	*<.*001	0.007	4.66 ± 1.72	*<.*001
No (Ref.)	3158 (76.5)		–	–		–	–		–	–		–	–
**Medical treatment in the past 14 days**													
Yes	342 (8.3)	0.009	3.68 ± 1.19	*<.*001	0.010	2.98 ± 0.92	*<.*001	0.007	3.21 ± 1.13	*<.*001	0.004	5.59 ± 2.65	*.*010
No (Ref.)	3784 (91.7)		–	–		–	–		–	–		–	–
**Direct contact with individual in the past 14 days with confirmed infection**													
Yes	107 (2.6)	0.000	1.41 ± 2.08	.19	0.002	2.36 ± 1.61	*.*004	0.001	1.90 ± 1.97	.06	0.000	3.61 ± 4.61	.12
No (Ref.)	4019 (97.4)		–	–		–	–		–	–		–	–
**Indirect contact with individual in the past 14 days with confirmed infection**													
Yes	202 (7.3)	0.003	2.25 ± 1.27	*<.*001	0.003	1.74 ± 0.98	*<.*001	0.003	2.05 ± 1.20	*<.*001	0.002	3.71 ± 2.81	*<.*01
No (Ref.)	3824 (92.7)		–	–		–	–		–	–		–	–
**Contact with individual with suspected infection**													
Yes	544 (13.2)	0.004	2.02 ± 0.97	*<.*001	0.004	1.60 ± 0.75	*<.*001	0.002	1.31 ± 0.92	*<.*001	0.003	4.11 ± 2.16	*<.*001
No (Ref.)	3582 (86.8)		–	–		–	–		–	–		–	–
**Contact with infectious materials individual**													
Yes	1472 (35.7)	0.001	0.79 ± 0.69	*.*024	0.003	0.88 ± 0.53	*.*001	0.001	0.85 ± 0.65	*.*011	0.000	0.80 ± 1.53	.31
No (Ref.)	2654 (64.3)		–	–		–	–		–	–		–	–
**Hospitalization in the past 14 days**													
Yes	17 (0.4)	0.001	3.91 ± 5.16	.14	0.000	2.59 ± 3.99	.20	0.001	4.20 ± 4.89	.09	0.000	6.55 ± 11.44	.26
No (Ref.)	4109 (99.6)		–	–		–	–		–	–		–	–
**Testing for COVID-19 in the past 14 days**													
Yes	62 (1.5)	0.003	4.49 ± 2.71	*<.*01	0.001	2.20 ± 2.10	*.*041	0.002	3.86 ± 2.57	*.*003	0.000	3.48 ± 6.03	.26
No (Ref.)	4064 (98.5)		–	–		–	–		–	–		–	–
**Quarantine in the past 14 days**													
Yes	177 (4.3)	0.003	2.80 ± 1.63	*<.*001	0.005	3.04 ± 1.26	*<.*001	0.003	2.82 ± 1.54	*<.*001	0.003	6.69 ± 3.61	*<.*001
No (Ref.)	3949 (95.7)		–	–		–	–		–	–		–	–

*Notes.* Descriptive statistics are presented as mean and standard deviations in parentheses for continuous predictor variables or n (%) for categorical predictor variables

In the last two weeks, 8.3% of the participants reported to have received medical treatment, 2.6% reported direct and 7.3% indirect contact with individuals with confirmed COVID-19 infection. Moreover, 13.2% reported contact with individuals with suspected COVID-19 infection, 35.7% contact with infected materials, and 0.4% had been admitted to the hospital. Only 1.5% had been tested for COVID-19 and 4.3% reported being under quarantine by a health authority. Medical treatment within the last 14 days was significantly associated with higher stress (*B* = 3.68, 95% *CI* = 2.49, 4.87, *p < .*001), anxiety (*B* = 2.98, 95% *CI* = 2.06, 3.90, *p < .*001), depression (*B* = 3.21, 95% *CI* = 2.08, 4.34, *p < .*001) and higher scores in the IES-R (*B* = 5.59, 95% *CI* = 2.94, 8.24, *p < .*001). Indirect contact with individuals with a confirmed COVID-19 infection and contact with an individual with suspected infection was significantly associated with higher stress (indirect contact: *B* = 2.25, 95% *CI* = 0.98, 3.52, *p < .*001; suspected contact: *B* = 2.02, 95% *CI* = 1.05, 2.99, *p < .*001), anxiety (indirect contact: *B* = 1.74, 95% *CI* = 0.76, 2.72, *p < .*001; suspected contact: *B* = 1.60, 95% *CI* = 0.85, 3.35, *p < .*001), depression (indirect contact: *B* = 2.05, 95% *CI* = 0.85, 3.25, *p < .*001; suspected contact: *B* = 1.31, 95% *CI* = 0.38, 2.23; *p = .*005) and higher scores in the IES-R (indirect contact: *B* = 3.71, 95% *CI* = 0.90, 6.52, *p = .*010; suspected contact: *B* = 4.11, 95% *CI* = 1.95, 6.27, *p < .*001). In contrast, direct contact with an individual with confirmed infection was significantly associated with anxiety (*B* = 2.36, 95% *CI* = 0.75, 3.97, *p = .*004), but not with stress, depression or the IES-R. Contact with potentially infectious material was significantly positively associated with stress (*B* = 0.79, 95% *CI* = 0.10, 1.48, *p = .*025), anxiety (*B* = 0.88, 95% *CI* = 0.35, 1.41, *p < .*001) and depression (*B* = 0.85, 95% *CI* = 0.20, 1.50, *p = .*011). Having been tested for COVID-19 was significantly associated with stress (*B* = 4.49, 95% *CI* = 1.78, 7.20, *p = .*001), anxiety (*B* = 2.20, 95% *CI* = 0.10, 4.30, *p = .*041) and depression (*B* = 3.86, 95% *CI* = 1.29, 6.43, *p = .*003). Being under quarantine within the last 14 days was significantly associated with more stress (*B* = 2.80, 95% *CI* = 1.57, 4.43, *p < .*001), anxiety (*B* = 3.04, 95% *CI* = 1.78, 4.10, *p < .*001), depression (*B* = 2.82, 95% *CI* = 1.28, 4.36, *p < .*001) and higher scores in the IES-R (*B* = 6.69, 95% *CI* = 3.07, 10.3, *p < .*001).

### Associations with virus-specific knowledge and concerns

The majority of the participants were aware of the increase of the number of infected individuals (99.7%), the number of deaths (99.1%) and the number of recovered individuals (87.7%). The knowledge about the increase in the number of recovered individuals and infections was significantly associated with lower stress (recovered: *B* = − 1.71, 95% *CI* = − 2.72, − 0.70, *p < .*001; infected: *B* = − 6.30, 95% *CI* = − 11.98, − 0.62, *p < .*001), anxiety (recovered: *B* = − 1.73, 95% *CI* = − 2.51, − 0.95, *p < .*001; infected: *B* = − 7.89, 95% *CI* = − 12.28, − 3.50, *p < .*001), depression (recovered: *B* = − 2.04, 95% *CI* = − 2.99, − 1.09, *p < .*001; infected: *B* = − 7.58, 95% *CI* = − 12.96, − 2.20, *p < .*001) and lower scores in the IES-R (recovered: *B* = − 4.36, 95% *CI* = − 6.59, − 2.13, *p < .*001; infected: *B* = − 12.76, 95% *CI* = − 25.36, − 0.16, *p < .*001). Further, the belief that COVID-19 cannot be transmitted via air, was significantly associated with lower stress (*B* = − 2.17, 95% *CI* = − 2.94, − 1.4, *p < .*001), anxiety (*B* = − 1.45, 95% *CI* = − 2.05, − 0.85, *p < .*001), depression (*B* = − 1.80, 95% *CI* = − 2.53; − 1.07, *p < .*001) and lower scores in the IES-R (*B* = − 3.74, 95% *CI* = − 5.46, − 2.02, *p < .*001).

The most prominent source of health information about COVID-19 was the internet (56.3%), followed by TV (30.3%) and radio (7.3). Internet as preferred source of information was significantly associated with higher stress (*B* = 1.37, 95% *CI* = − 0.89, 3.63) and depression (*B* 1.04, 95% *CI* = − 0.82, 2.90) as compared to the reference category “TV”. Most of the respondents (84.9%) were highly satisfied or somewhat satisfied with the available health information. Satisfaction with the health information was significantly associated with lower anxiety (*B* = − 2.44, 95% *CI* = − 4.21, − 0.67, *p = .*007), while dissatisfaction with the provided information was associated with higher stress (*B* = 4.26, 95% *CI* = 0.93, 7.59, *p = .*012).

Most of the participants stated that they are very confident (20.8%) or confident (58.6%) regarding the diagnostic capabilities of the health system, while 18.5%, respectively, 2.1% were rather not confident or not confident at all. Less confidence in the doctor’s ability to diagnose COVID-19 was significantly related to higher stress (*B* = − 2.72, 95% *CI* = − 3.19, − 2.25, *p < .*001), anxiety (*B* = − 1.99, 95% *CI* = − 2.36, − 1.62, *p < .*001), depression (*B* = − 2.70, 95% *CI* = − 3.15, − 2.25, *p < .*001) and a higher IES-R score (*B* = − 4.77, 95% *CI* = − 5.82, − 3.72, *p < .*001). 71.5% were very worried or somewhat worried about other family members getting COVID-19, while 41.6% of the respondents were very worried or somewhat worried about their children getting infected. High levels of concern about other family members or children were significantly associated with higher stress (family members: *B* = 5.39, 95% *CI* = 3.43, 7.35, *p < .*001; children: *B* = 5.21, 95% *CI* = 3.85, 6.57, *p < .*001), anxiety (family members: *B* = 4.16, 95% *CI* = 2.64, 5.68, *p < .*001; children: *B* = 4.48, 95% *CI* = 3.43, 5.53, *p < .*001), depression (family members: *B* = 2.97, 95% *CI* = 1.09, 4.85, *p = .*002; children: *B* = 4.48, 95% *CI* = 3.43, 5.53, *p < .*001), and more psychological impact of the outbreak (family members: *B* = 10.47, 95% *CI* = 6.12, 14.82, *p < .*001; children: *B* = 12.10, 95% *CI* = 9.09, 15.11, *p < .*001).

Almost half of the participants (49.8%) thought that an own infection was likely or very likely, but the majority (92.2%) believed that it was very likely or somewhat likely to survive a COVID-19 infection. Higher perceived likelihood of being infected with COVID-19 was significantly associated with higher stress (*B* = 2.94, 95% *CI* = 1.49, 4.39, *p < .*001) and depression (*B* = 2.40, 95% *CI* = 1.02, 3.78, *p < .*001), while a higher perceived likelihood of surviving COVID-19 infection was significantly associated with less stress (*B* = − 4.71, 95% *CI* = − 6.26, − 3.16, *p < .*001), anxiety (*B* = − 5.04, 95% *CI* = − 6.59, − 4.21, *p < .*001), depression (*B* = − 4.18, 95% *CI* = − 5.65, 2.71, *p < .*001) and impact of event (*B* = − 14.35, 95% *CI* = − 17.76, − 10.94, *p < .*001). For details, see Table 3.

**Table 3:** Linear regression results of variables related to concerns and knowledge associated with the psychological impact of the 2020 COVID-19 outbreak measured with DASS-Subscales and the IES-R

***Variables***	***N (%)***	Stress			Anxiety			Depression			Impact of Event		
		*R*^*2*^	*B (*95% *CI)*	*p*	*R*^*2*^	*B (*95% *CI)*	*p*	*R*^*2*^	*B (*95% *CI)*	*p*	*R*^*2*^	*B (*95% *CI)*	*p*
**Transmission via droplets**													
Yes	4077 (98.8)		2.41 ± 3.60	.98		−0.03 ± 2.79	.98		−0.92 ± 3.41	.60		6.85 ± 7.99	.09
No	14 (0.3)	0.000	6.26 ± 6.71	.06	0.000	5.00 ± 5.20	.06	0.001	4.51 ± 6.36	.16	0.000	15.53 ± 14.89	*<.*05
Unsure (Ref.)	35 (0.8)		–	–		–	–		–	–		–	–
**Transmission via contaminated objects**													
Yes	2767 (67.1)		0.67 ± 0.75	.08		0.57 ± 0.58	.06		0.60 ± 0.71	.10		0.11 ± 1.67	.90
No	236 (5.7)	0.001	−1.09 ± 1.52	.16	0.002	− 1.05 ± 1.18	.08	0.001	−0.97 ± 1.44	.19	0.000	−3.16 ± 3.37	.07
Unsure (Ref.)	1123 (27.2)		–	–		–	–		–	–		–	–
**Transmission via air**													
Yes	978 (23.7)		0.27 ± 0.91	.55		0.60 ± 0.70	.10		0.21 ± 0.86	.64		0.70 ± 2.01	.49
No	1929 (46.8)	0.004	−2.17 ± 0.77	*<.*001^a^	0.006	−1.45 ± 0.60	*<.*001^a^	0.003	−1.80 ± 0.73	*<.*001^a^	0.003	−3.74 ± 1.72	*<.*001^a^
Unsure (Ref.)	1219 (29.5)		–	–		–	–		–	–		–	–
**Knowledge about increasing infections**													
Yes	4112 (99.7)	0.001	−6.30 ± 5.68	*.*030	0.003	−7.89 ± 4.39	*<.*001	0.002	−7.58 ± 5.38	*.*006	0.001	−12.76^a^ ± 12.60 12.60	*.*047
No (Ref.)	14 (0.3)		–	–		–	–		–	–		–	–
**Knowledge about increasing deaths**													
Yes	4088 (99.1)	0.000	−1.86 ± 3.46	.29	0.000	−1.54 ± 2.68	.26	0.000	−2.36 ± 3.28	.16	0.000	−0.09 ± 7.67	.98
No (Ref.)	38 (0.9)		–	–		–	–		–	–			–
**Knowledge about increasing recoveries**													
Yes	3619 (87.7)	0.003	−1.71 ± 1.01	*<.*001	0.005	−1.73 ± 0.78	*<.*001	0.004	−2.04 ± 0.95	*<.*001	0.004	−4.36 ± 2.23	*<.*001
No (Ref.)	507 (12.3)		–	–		–	–		–	–		–	–
**Main source of information**													
Internet	2324 (56.3)		1.37 ± −2.26	*<.*001^a^		0.30 ± −0.73	.30		1.04 ± −1.86	*<.*001^a^		−0.21 ± 1.65	.80
Radio	303 (7.3)		0.03 ± −0.01	.97		0.58 ± −0.50	.28		0.12 ± −0.07	.85		0.99 ± 3.01	.52
Family/Friends	78 (1.9)	0.005	0.91 ± 0.19	.47	0.003	1.23 ± −0.03	.21	0.004	1.28 ± 0.21	.28	0.001	1.41 ± 5.49	.61
Others (e.g. newspapers)	169 (4.1)		−1.93 ± 0.25	*.*029		−1.75 ± 0.80	*.*011^a^		−1.40 ± 0.27	.10		−4.15 ± 3.86	*.*035
TV (Ref.)	1252 (30.3)		–	–		–	–		–	–		–	–
**Satisfaction with information**													
Very satisfied	1696 (41.1)		−1.92 ± 2.28	.10		−2.44 ± 1.77	*.*007^a^		−2.56 ± 2.16	*.*020		−3.84 ± 5.08	.14
Somewhat satisfied	1974 (47.8)		0.26 ± 2.27	.82		−1.28 ± 1.76	.16		−0.70 ± 2.15	.53		−1.53 ± 5.06	.55
Not very satisfied	294 (7.1)	0.012	2.00 ± 2.54	.12	0.011	0.18 ± 1.97	.86	0.015	1.19 ± 2.41	.33	0.005	2.07 ± 5.66	.47
Not satisfied at all	72 (1.7)		4.26 ± 3.33	*.*012^a^		1.17 ± 2.59	.38		2.34 ± 3.16	.15		1.96 ± 7.43	.61
Do not know (Ref.)	90 (2.2)		–	–		–	–		–	–		–	–
**Confidence in diagnosis capability**													
**4-point Likert scale**	2.98 (0.70)	0.030	−2.72 ± 0.47	*<.*001	0.027	−1.99 ± 0.37	*<.*001	0.033	−2.70 ± 0.45	*<.*001	0.019	−4.77 ± 1.05	*<.*001
(Continuous)			–	–		–	–		–	–		–	–
**Likelihood of own infection**													
Very likely	467 (11.3)		2.94 ± 1.45	*<.*001^a^		1.24 ± 1.12	.030		2.40 ± 1.38	*.*001^a^		2.29 ± 3.23	.16
Somewhat likely	1587 (38.5)		−0.36 ± 1.20	.56		−0.98 ± 0.93	*.*039		−0.18 ± 1.14	.75		−2.59 ± 2.67	.06
Not very likely	1344 (32.6)	0.025	−1.95 ± 1.22	*.*002^a^	0.020	−2.08 ± 0.95	*<.*001^a^	0.015	−1.30 ± 1.16	*.*028	0.014	−4.29 ± 2.72	*.*002^a^
Not likely at all	348 (8.4)		−3.75 ± 1.56	*<.*001^a^		−3.23 ± 1.21	*<.*001^a^		−2.48 ± 1.48	*.*001^a^		−9.43 ± 3.47	*<.*001^a^
Do not know (Ref.)	380 (9.2)		–	–		–	–		–	–		–	–
**Likelihood of surviving**													
Very likely	2169 (52.6)		−4.71 ± 1.55	*<.*001^a^		−5.04 ± 1.19	*<.*001^a^		−4.18 ± 1.47	*<.*001^a^		−14.35 ± 3.41	*<.*001^a^
Somewhat likely	1632 (39.6)		−2.61 ± 1.57	*.*001^a^		−3.05 ± 1.21	*<.*001^a^		−2.40 ± 1.49	*.*002^a^		−8.58 ± 3.46	*<.*001^a^
Not very likely	92 (2.2)	0.018	0.78 ± 2.65	.56	0.034	1.08 ± 2.03	.30	0.019	1.83 ± 2.51	.15	0.031	−0.33 ± 5.83	.91
Not likely at all	31 (0.8)		−0.78 ± 4.06	.71		0.11 ± 3.12	.95		1.26 ± 3.84	.52		0.80 ± 8.95	.86
Do not know (Ref.)	202 (4.9)		–	–		–	–		–	–		–	–
**Concerns about family members**													
Very worried	1215 (29.4)		5.39 ± 1.96	*<.*001^a^		4.16 ± 1.52	*<.*001^a^		2.97 ± 1.88	*.*002^a^		10.47 ± 4.35	*<.*001^a^
Somewhat worried	1737 (42.1)		0.89 ± 1.93	.37		0.62 ± 1.50	.42		−0.83 ± 1.86	.38		0.51 ± 4.29	.82
Not very worried	871 (21.1)	0.068	−2.06 ± 2.00	*.*043	0.061	−1.36 ± 1.55	.09	0.042	−2.49 ± 1.92	*.*011^a^	0.069	−6.12 ± 4.42	*.*007^a^
Not worried at all	183 (4.4)		−2.36 ± 2.41	.05		−0.70 ± 1.87	.46		−2.46 ± 2.31	*.*037		−7.63 ± 5.34	*.*005^a^
Do not know (Ref.)	120 (2.9)		–	–		–	–		–	–		–	–
**Concerns about children**													
Very worried	262 (18.1)		5.21 ± 1.36	*<.*001^a^		4.48 ± 1.05	*<.*001^a^		2.53 ± 1.29	*<.*001^a^		12.10 ± 3.01	*<.*001^a^
Somewhat worried	340 (23.5)		1.47 ± 1.21	*.*017		0.43 ± 0.94	.36		−1.38 ± 1.15	*.*019^a^		4.18 ± 2.68	*.*002^a^
Not very worried	645 (44.5)	0.017	−0.62 ± 0.92	.19	0.024	−1.49 ± 0.71	*<.*001^a^	0.014	−2.24 ± 0.88	*<.*001^a^	0.023	−3.61 ± 2.04	*<.*001^a^
Not worried at all	184 (12.7)		−1.56 ± 1.60	.06		−1.09 ± 1.24	.09		−2.39 ± 1.52	*.*02^a^		−3.96 ± 3.55	*.*029^a^
Do not know (Ref.)	17 (1.2)		–	–		–	–		–	–		–	–

*Notes.* Descriptive statistics are presented as mean and standard deviations in parentheses for continuous predictor variables or n (%) for categorical predictor variables

^a^significant after Bonferroni Holm adjustment for multiple comparisons

### Associations with precautionary measures

97.3% of the participants stated, that they were mostly or always washing their hands thoroughly, 81.6% were mostly or always washing their hands immediately after touching a potentially infectious object, 78.68% were mostly or always covering their mouth when sneezing or coughing, 66.4% were mostly or always washing their hands immediately after sneezing or coughing, 64.6% mostly or always avoided sharing utensils, 20.6% reported that they are mostly or always wearing gloves while shopping and 3.7% reported to frequently wear face masks. Covering the mouth while coughing and sneezing was significantly associated with higher stress (*B* = 0.35, 95% *CI* = 0.08, 0.62, *p = .*011), anxiety (*B* = 0.32, 95% *CI* = 0.11, 0.53, *p < .*002), and higher scores in the IES-R (*B* = 1.77, 95% *CI* = 1.17, 2.37, *p < .*001). Washing hands thoroughly was significantly associated with lower depression (*B* = − 0.62, 95% *CI* = − 1.20, − 0.04, *p = .*036) and higher scores in the IES-R (*B* = 2.89, 95% *CI* = 1.53, 4.25, *p < .*001). Washing hands immediately after coughing or sneezing and washing hands after touching contaminated objects was significantly associated with higher anxiety (coughing/sneezing: *B* = 0.43, 95% *CI* = 0.21, 0.65, *p < .*001; touching objects: *B* = 0.53, 95% *CI* = 0.29, 0.77, *p < .*001) and higher scores in the IES-R (coughing/sneezing: *B* = 1.96, 95% *CI* = 1.34, 2.58, *p < .*001; touching objects: *B* = 2.25, 95% *CI* = 1.57, 2.93, *p < .*001). Wearing masks and gloves was significantly associated with higher stress (masks: *B* = 0.74, 95% *CI* = 0.32, 1.16, *p < .*001; gloves: *B* = 0.61, 95% *CI* = 0.39, 0.83, *p < .*001), anxiety (masks: *B* = 0.83, 95% *CI* = 0.51, 1.15, *p < .*001; gloves: *B* = 0.63, 95% *CI* = 0.46, 0.80, *p < .*001), depression (only wearing gloves: *B* = 0.46, 95% *CI* = 0.25, 0.67, *p < .*001), and higher scores in the IES-R (masks: *B* = 2.28, 95% *CI* = 1.36, 3.20, *p < .*001; gloves: *B* = 2.11, 95% *CI* = 1.63, 2.59, *p < .*001).

The majority (83.1%) of the participants continued their physical activity during the last 14 days. Physical activity was significantly associated with lower stress (*B* = − 0.90, 95% *CI* = − 1.13, − 0.67, *p < .*001), anxiety (*B* = − 0.65, 95% *CI* = − 0.83, − 0.47, *p < .*001), depression (*B* = − 0.89, 95% *CI* = − 1.11, − 0.67, *p < .*001) and lower scores in the IES-R (*B* = − 1.50, 95% *CI* = − 2.02, − 0.98, *p < .*001). 15.4% of the individuals always or often felt that too much unnecessary worry had been made about COVID-19. Interestingly, this belief was associated with higher stress (*B* = 0.81, 95% *CI* = 0.50, 1.12, *p < .*001), anxiety (*B* = 0.51, 95% *CI* = 0.27, 0.75, *p < .*001), depression (*B* = 0.90, 95% *CI* = 0.61, 1.19, *p < .*001) and higher scores in the IES-R (*B* = 1.16, 95% *CI* = 0.48, 1.84, *p < .*001). For details see Table 4.

**Table 4:** Linear regression results of precautionary measures during the past 14 day associated with the psychological impact of the 2020 COVID-19 outbreak measured with DASS-Subscales and the IES-R

***Variables***	***M (SD)***	Stress			Anxiety			Depression			Impact of Event		
		*R*^*2*^	*B (*95% *CI)*	*p*	*R*^*2*^	*B (*95% *CI)*	*p*	*R*^*2*^	*B (*95% *CI)*	*p*	*R*^*2*^	*B (*95% *CI)*	*p*
**Covering mouth while coughing or sneezing**													
**5-point Likert scale** (1 = never to 5 = always; continuous)	4.02 (1.23)	0.002	0.35 ± 0.27	*.*011	0.002	0.32 ± 0.21	*.*002	0.000	0.02 ± 0.26	.90	0.008	1.77 ± 0.60	*<.*001
**Avoiding sharing utensils**													
**5-point Likert scale** (1 = never to 5 = always; continuous)	3.62 (1.49)	0.000	0.16 ± 0.22	.15	0.001	0.14 ± 0.17	.10	0.000	0.12 ± 0.21	.26	0.000	0.26 ± 0.49	.30
**Thorough hand washing**													
**5-point Likert scale** (1 = never to 5 = always; continuous)	4.72 (0.54)	0.000	0.19 ± 0.61	.54	0.001	0.38 ± 0.47	.11	0.001	−0.62 ± 0.58	*.*036	0.004	2.89 ± 1.36	*<.*001
**Washing hands immediately after coughing or sneezing**													
**5-point Likert scale** (1 = never to 5 = always; continuous)	3.78 (1.18)	0.000	0.19 ± 0.28	.19	0.004	0.43 ± 0.22	*<.*001	0.000	−0.19 ± 0.27	.17	0.009	1.96 ± 0.62	*<.*001
**Wearing a face mask**													
**5-point Likert scale** (1 = never to 5 = always; continuous)	1.30 (0.79)	0.003	0.74 ± 0.42	*<.*001	0.006	0.83 ± 0.32	*<.*001	0.001	0.36 ± 0.40	.08	0.006	2.28 ± 0.92	*<.*001
**Wearing gloves while shopping**													
**5-point Likert scale** (1 = never to 5 = always; continuous)	1.98 (1.50)	0.007	0.61 ± 0.22	*<.*001	0.013	0.63 ± 0.17	*<.*001	0.004	0.46 ± 0.21	*<.*001	0.017	2.11 ± 0.48	*<.*001
**Washing hands after contact with potentially infectious objects**													
**5-point Likert scale** (1 = never to 5 = always; continuous)	4.28 (1.07)	0.003	0.56 ± 0.31	*<.*001	0.005	0.53 ± 0.24	*<.*001	0.000	0.20 ± 0.29	.18	0.010	2.25 ± 0.68	*<.*001
**Physical activity**													
**5-point Likert scale** (1 = never to 5 = always; continuous)	3.19 (1.41)	0.014	−0.90 ± 0.23	*<.*001	0.012	−0.65 ± 0.18	*<.*001	0.015	−0.89 ± 0.22	*<.*001	0.008	−1.50 ± 0.52	*<.*001
**Feeling that too much unnecessary worry has been made**													
**5-point Likert scale** (1 = never to 5 = always; continuous)	2.44 (1.07)	0.014	0.81 ± 0.31	*<.*001	0.017	0.51 ± 0.24	*<.*001	0.019	0.90 ± 0.29	*<.*001	0.008	1.16 ± 0.68	*<.*001

### Need for additional health information

**Fig. 2:**
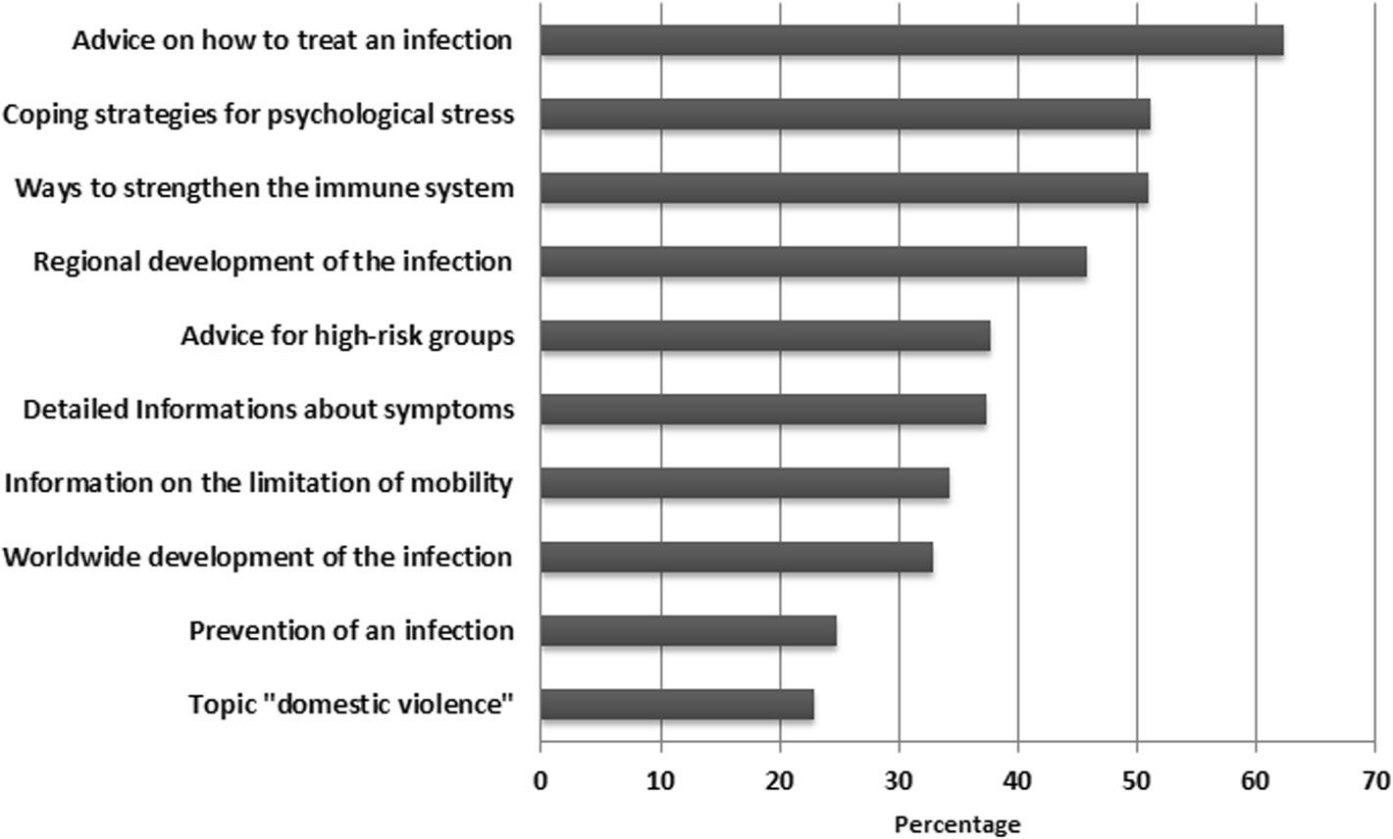
Need for essential further information and advice. Results of the item: “What information or advice are you missing in particular?”

Almost all participants asked for additional information about COVID-19. Most frequently mentioned were advices on how to treat an infection (62.4%), more information about coping strategies for psychological stress (51.1%), ways to strengthen the immune system (50.9%) and information about the regional development of the infection (46%). 24.8% requested detailed information about how to prevent an infection and 23% of the individuals needed further information for victims of domestic violence. For details, see Fig. 2. The need for the respective information was significantly associated with higher stress, anxiety, depression and psychological impact of the event (data not shown).

## Discussion

This study aimed to examine the psychosocial correlates during the initial state of COVID-19 pandemic in Austria, using a cross-sectional study design. 43.3% of the participants rated the psychological impact of the COVID-19 outbreak as moderate (5.6%) or severe (37.7%). 26.5% reported moderate (13.3%) to severe (13.2%) depression; 20.3% moderate (8.9%) to severe (11.4%) anxiety and 21.2% were considered to suffer from moderate (10.5%) or severe stress (10.7%). As compared to a recently published Chinese study on the psychological correlates of the COVID-19 outbreak [4], which was conducted at the same developmental stage of the outbreak in Austria, several differences emerged. The Chinese study population reported a higher psychological impact of the COVID-19 pandemic as compared to the Austrian study population. Furthermore, significant differences could be observed in depression, anxiety and stress. The Austrian study population showed higher values in all three categories as compared to the Chinese sample. One possible explanation for these findings could be the low level of information, which was available at the time of the COVID-19 outbreak in China and the assumption that COVID-19 virus was seen as a regional Chinese problem. At the time of the survey in Austria, COVID-19 has already been classified as a pandemic, which could have led to increased stress, depression and anxiety among the Austrian population due to its global spread and the perceived threat and the feeling of insecurity. For example, there is evidence that the feeling of being unsafe is associated with an increased level of psychological distress [11]. These increased values could in turn have been the reason for the Austrian population’s compliance with the government’s measures.

Results showed that 56.3% of the participants used the internet as main source of information. This is in line with recent COVID-19 research from China [4]. Interestingly, using the internet was positively associated with depression, anxiety and stress. This is not surprising as, on the internet or in social media in particular, information is available which is not necessarily based on well-founded facts. This could lead to confusion and uncertainty among individuals regarding the health policies taken to avoid the spreading of the COVID-19 virus. Relatedly, downplaying and unacceptability of the seriousness of the COVID-19 pandemic was associated with depression, stress, and anxiety. In contrast, being aware of the hazards of the COVID-19 pandemic (e.g., increasing numbers of infections, transmission routes, etc.) was associated with decreased depression, stress and anxiety. On the other hand, elevated levels of anxiety, depression, and stress could also trigger internet use in order to get more information and counter uncertainty.

15.4% of the Austrian population were downplaying the seriousness of this pandemic. Interestingly, this belief was associated with higher stress, anxiety, depression and psychological impact of the event. This could be an indicator for an emotion regulation strategy like, for example, suppression of emotions, which is associated with increased values of depression, anxiety and stress [12–14].

In contrast to the findings in China [4], within the Austrian study population most of the precautionary measures like, for example, washing hands or covering the mouth when sneezing or coughing, were associated with higher stress, anxiety, depression and/or psychological impact of event. The increasing awareness of precautions to avoid infection with the virus in the context of the COVID-19 pandemic may have led to insecurity and subsequently to increased anxiety, stress and depression [11]. On the other hand, elevated levels of anxiety, depression and stress could have driven the uptake of preventive efforts. Specifically, according to several theories of health behavior, perceived threat and vulnerability could trigger behavior change [15–17].

Results of the present study could help defining psychological risk groups that are particularly affected by the COVID-19 pandemic. In line with previous studies from China [4, 18, 19], women and students seem to experience elevated psychological symptoms related to this virus pandemic as compared to men and employed individuals. The sex difference is in line with evidence suggesting that women are more vulnerable to stress and more likely to develop posttraumatic stress disorder than men [20–22]. Furthermore, in this exceptional situation, women are faced with additional tasks, thus possibly imposing further burden. Specifically, in Austria, 48.8% of the women in our sample worked via home office. Many of them have to support their children in learning, since all schools were shut down. Hence, supporting women in this situation might be especially important.

Students and unemployed individuals were also found to experience a comparably strong psychological impact of the outbreak of COVID-19. Students were particularly affected by the COVID-19 pandemic as there were no lectures, examination regulations were not clarified and thus students had to fear for their scholarships. In Austria, the first intervention for students, which was implemented after the survey, was a comparably fast transition from classroom teaching to digital/virtual teaching. Students, who needed more supervision were offered video conferences with their supervisors. Additionally, two weeks after the universities were closed, the Austrian government declared the summer semester as “neutral” semester, which means that students do not lose their financial aid. Furthermore, the government aimed to provide detailed information about the further course of the semester as fast as possible. An important question that has not yet been clarified is whether and if so, how written examinations can take place this semester. There is evidence that unemployment is associated with decreased mental health [23, 24]. This psychological strain may have been further increased by the COVID-19 pandemic, as prescribed courses cannot be held and thus the conditions defined by the employment office cannot be fulfilled. This increases concerns about the future and financial security of this group. The Austrian government implemented special aid programs for people who have lost their jobs due to the crisis. In order to keep the rise in unemployment during the COVID-19 pandemic as low as possible, companies were offered various financial aid packages by the authorities. Further follow-up studies are needed to clarify whether these quick-acting measures will impact psychosocial health of this risk group.

Interestingly, the group of self-employed individuals apparently experienced less psychological impact during the COVID-19 outbreak as compared to other professions. This could possibly be explained such that self-employed individuals are often confronted with uncertainties and have learnt to cope with it. Results of previous studies [4, 18], suggesting a higher level of education to be associated with higher psychological distress could not be confirmed. Rather, the findings of the present study showed exactly the opposite and are in line with results of Liu et al. (2020). Higher educated individuals experienced a lower psychological impact during the COVOD-19 pandemic. Liu et al. (2020) argued that those with lower education level know less about coping strategies during acute stress events, which in turn lead to decreased adaptability and resilience as compared to individuals with higher formal education. Another possible explanation could be that higher educated people search more selectively for COVID-19 information and can possibly better distinguish between evidence-based and erroneous/biased information.

Further findings showed that the current state of health was associated with psychological distress, thus supporting previous research [26, 27]. Individuals who reported physical symptoms like cough, breathing problems or those who rated their own state of health as bad experienced more stress, anxiety, and depression. Reports from Austria showed that the counseling centers for mental health problems were completely overloaded during the initial outspread of COVID-19, thus calling for an increase of personnel resources in this area. It could also be shown that participants needed more information about how to treat an infection themselves, and that this need was significantly associated with higher stress, anxiety, depression and psychological impact of the event.

Of note, there is evidence that physical activity has a positive effect on mental health [28–31]. Results showed that this positive association could also be observed in this exceptional situation. Physical activity was associated with less anxiety, depression and stress. Although the findings are merely correlational, thus precluding any causal effects (e.g., higher anxiety and depression could also inhibit physical activity), based on previous experimental evidence [32] the findings are compatible with a mental health-protective role of physical activity. The question arises under which conditions physical activity could be encouraged in such an exceptional situation, especially since the strict regiments in many countries do not allow the public to practice sports outside. To the contrary, many parks and green spaces in cities are closed due to fear of a further spread of the virus. In Austria, for example, the government did not generally prohibit the practice of physical activities outdoors, but encourages it in compliance with clearly defined rules, such as keeping distance from strangers or people who do not live in the same household.

This study has several limitations that need to be discussed: First, this study used a cross-sectional design, correlational design, which precludes any causal inferences. Strictly speaking, we cannot conclude that the COVID-19 pandemic impacted psychosocial well-being, albeit it seems reasonable to interpret the data in such way. Relatedly, because of the cross-sectional design psychological associations with the COVID-19 pandemic were related to a certain time point, which in this case was the initial phase of the outbreak in Austria. Hence, findings reflect a momentary state of affairs, which could change rapidly, given the dynamic of the COVID-19 pandemic. Relatedly, associations among measures (e.g., health preventive behaviors and psychological symptoms) could be bidirectional. Second, there was a different distribution between men and women with men being underrepresented and an oversampling of individuals with academic background. Hence, findings cannot be regarded representative for the population in Austria.

## Conclusion

43.3% of the study sample rated the psychological impact of the COVID-19 outbreak as moderate (5.6%) or severe (37.7%). Similarities and differences could be found in the Chinese and the Austrian study population related to the psychological impact on COVID-19, manifested on the level of depression, anxiety and stress. Chinese study population showed higher psychological impact of the COVID-19 pandemic compared to the Austrian participants, whereas the Austrian study population reported higher scores in depression, anxiety and stress as compared to the Chinese study participants. Furthermore, precautionary measures were associated with higher values of depression, anxiety and stress in the Austrian study population compared to the Chinese study population. Individuals with high educational background within the Chinese population reported higher values in depression, anxiety and stress as compared to the Austrian participants. Similarities between the Chinese and the Austrian study population could be found in the identification of potentially vulnerable groups related to the COVID-19 pandemic, suggesting that, in both study populations, women and students experienced elevated psychological symptoms as compared to other professional groups. In Austria, also unemployed individuals seemed to show elevated psychological symptoms.

The findings of the present study may lead to the following recommendations: (1) early and rapid identification of risk groups, like women, students, unemployed in order to provide assistance; (2) downplaying and unacceptability of the seriousness of the COVID-19 pandemic seems to be associated with depression, stress, and anxiety, which supports the necessity of evidence-based information of the seriousness of the situation and highlighting of the dangers of downplaying the seriousness of the pandemic; (3) provision of easily accessible information regarding coping strategies for psychological stress and advices on how to treat an infection yourself, since the need for the respective information was associated with higher psychological burden; (4) due to the high demand for psychological counselling, staff reinforcement is needed in this area; (5) using the internet was highly associated with depression, anxiety and stress and, which highlights the necessity of education and information on the risks of using social media to obtain information especially in this context.

The present findings provide an orientation for the identification of psychologically vulnerable groups and for the development of psychological interventions. Further, this study may contribute to international comparison. Especially the possibility to compare results from China with those of a western country are crucial since, to the author’s knowledge, studies on Asian populations are currently the only source of information regarding the psychological impact during the COVID-19 outbreak and transcultural differences in psychosocial functioning following major life events are likely. As this is the first study on the psychological correlates during the COVID-19 pandemic in a European country, it should serve as a basis for further research.

## Data Availability

Data are available upon request from the authors of the article.

## Abbreviations

COVID-19: Coronavirus SARS-COV2 disease 2019
SARS-CoV-2: Severe acute respiratory syndrome coronavirus 2
SARS: Severe acute respiratory syndrome
DASS-21: Depression, Anxiety and Stress Scale
IES-R: Impact of Event Scale-Revised
RS-11: Resilience Scale
WHO: World Health Organization
